# Tooth discoloration induced by endodontic sealers and cervical limit: 1-year in vitro evaluation

**DOI:** 10.1590/0103-6440202305552

**Published:** 2023-12-22

**Authors:** Isabella Marian Lena, Cheiene Deriê Roncaglio Bagnara, Juliana Estivalet Visentini, Carlos Eduardo Victor da Costa Ribeiro, Liliana Gressler May, Renata Dornelles Morgental

**Affiliations:** 1 Graduate Program in Dental Science; Federal University of Santa Maria(UFSM), Santa Maria, Rio Grande do Sul, Brazil.; 2 Federal University of Santa Maria(UFSM), Santa Maria, Rio Grande do Sul, Brazil.; 3 Department of Restorative Dentistry/ Graduate Program in Dental Science; Federal University of Santa Maria(UFSM), Santa Maria, Rio Grande do Sul, Brazil; 4 Department of Stomatology/ Graduate Program in Dental Science; Federal University of Santa Maria(UFSM), Santa Maria, Rio Grande do Sul, Brazill.

**Keywords:** Endodontic sealers, Calcium silicate-based sealers, Tooth discoloration, Spectrophotometric analysis

## Abstract

This laboratory study aimed to evaluate the influence of endodontic sealer and cervical limit of root filling on the discoloration of root canal treated teeth. Bovine incisors were randomly distributed into six experimental groups and control (n=21/group), according to the endodontic sealer used [AH Plus (AP); MTA Fillapex (MF) and Sealer Plus BC (SPB)] and the cervical limit of root filling [dental cervix (DC) or 2 mm in apical direction (2mm-AD)]. Tooth discoloration (ΔE) was evaluated by a digital spectrophotometer using the CIED2000 method. Color assessments were performed immediately before (baseline), 1 week, 1, 3, 6 months, and 1 year after obturation. Data were analyzed by ANOVA and Tukey’s post-hoc tests (α=5%). Teeth filled with the three sealers showed perceptible tooth discoloration (ΔE≥2.7) in 1 week, maintaining similar values over time. There was a significant difference between MF and SPB sealers in the 2mm-AD groups. In addition, 2mm-AD groups promoted significantly lower discoloration than DC groups for AH (3 months) and SPB (1 and 3 months) sealer,s. Teeth filled with AP, MF, and SPB sealers displayed discoloration from 1 week to one year, with differences between MF and SPB sealers. A cervical limit of filling material at 2 mm from the dental cervix seems more advisable, promoting lower crown discoloration.

## Resumo

O presente estudo laboratorial teve como objetivo avaliar a influência do cimento endodôntico e do limite cervical da obturação radicular na alteração de cor de dentes tratados endodonticamente. Incisivos bovinos foram distribuídos aleatoriamente em seis grupos experimentais e um controle (n=21/grupo), de acordo com o cimento endodôntico utilizado [AH Plus (AP); MTA Fillapex (MF) e Sealer Plus BC (SPB)] e o limite cervical da obturação [Colo dentário (CD) ou 2mm na direção apical (2mm-DA)]. A alteração de cor (ΔE) foi avaliada por um espectofotômetro digital usando o método CIED2000. As avaliações de cor foram realizadas imediatamente antes (*baseline*), 1 semana, 1, 3, 6 meses e 1 ano após a obturação. Os dados foram analisados pelos testes ANOVA e post-hoc de Tuckey (α=5%). Dentes obturados com os três cimentos apresentaram alteração de cor perceptível (ΔE≥2.7) em 1 semana, mantendo valores semelhantes ao longo do tempo. Houve uma diferença significativa entre os cimentos MF e SPB nos grupos 2mm-DA. Além disso, os grupos 2mm-DA promoveram uma alteração de cor significativamente menor do que o grupos CD para os cimentos AH (3 meses) e SPB (1 e 3 meses). Os dentes obturados com os cimentos AH, MF e SPB apresentaram alteração de cor entre 1 semana a um ano, com diferenças entre os cimentos MF e SPB. O limite cervical do material obturador a 2mm do colo dentário parece mais aconselhável, promovendo menor alteração de cor da coroa dentária.

## Introduction

Tooth discoloration that results from endodontic treatments is a recurring finding in dental practice and may represent an aesthetic problem, which can negatively impact the patient's quality of life ^1^. The major causes of tooth discoloration related to endodontic treatment are caused by remnants of necrotic pulp tissue, intracanal medicaments, irrigant solutions (e.g., interaction between sodium hypochlorite and chlorhexidine), and filling materials ^2^. A recurrent problem is the presence of remaining materials in the pulp chamber, which get dark over time. This darkened pigment can be transmitted through the hard tissue ^(^ ^3^. Furthermore, material particles can penetrate the dentinal tubules and cause discoloration in the long term ^4^. Several in v,itro studies have shown the discoloration potential of gutta-percha ^5^ ^,^ ^6^ ^)^ and endodontic sealers ^7^ ^,^ ^8^ ^,^ ^9^ ^,^ ^10^ ^,^ ^11^ ^,^ ^12^

The epoxy resin-based sealer AH Plus (Dentsply Sirona, Ballaigues, Switzerland) is the gold standard of this category of sealers and has good worldwide acceptance because of its great physicochemical properties ^13^. Previous investigations have already demonstrated that AH Plus achieved great color stability during six months ^14^ and one year ^15^. However, other authors reported a clinically perceptible color change in 10 days, increasing over time ^8^.

In the last decade, Mineral Trioxide Aggregate (MTA) based sealers have been developed to explore the favorable biological properties of the aggregate ^16^. However, there is evidence of the chromogenic potential of these bioactive materials ^2^. MTA Fillapex (Angelus, Londrina, Paraná, Brazil) was introduced to the market in 2011, containing salicylate resin, natural resin, bismuth oxide, and nanoparticulated silica ^16^. Published studies demonstrated, *in vitro* and *in vivo*, that MTA-based sealers can promote tooth discoloration in different experimental periods ^8^ ^,^ ^9^ ^,^ ^17^.

More recently, premixed calcium silicate-based endodontic sealers have been developed and received great attention from the scientific community mainly because of their biocompatibility and bioactivity ^18^. Regarding tooth discoloration, the bioceramic sealer iRoot SP (Innovative BioCeramics, Vancouver, Canada) showed similar behavior to AH Plus and MTA Fillapex during the first six months ^8^. Sealer Plus BC (MK Life, Porto Alegre, Rio Grande do Sul, Brazil) has been recently introduced in the market; thus, studies have yet to investigate its potential for tooth discoloration.

It is a clinical recommendation that the obturation cervical limit should be positioned close to the gingival margin, below the cementoenamel junction (CEJ) ^17^. Additionally, the pulp chamber must be cleaned with 95% ethanol ^10^. However, to our knowledge, there is only one study regarding the ideal obturation cervical limit to avoid or minimize the tooth discoloration caused by different endodontic sealers during root canal treatment ^17^, and none using premixed calcium silicate-based materials. In this context, the present *in vitro* study aimed to evaluate the influence of the endodontic sealer (AH Plus, MTA Fillapex, or Sealer Plus BC) and the cervical limit (dental cervix or 2mm in apical direction) on the tooth discoloration of bovine root canal treated teeth. The null hypothesis (H_o_) was that none of these sealers would cause perceptible tooth discoloration over the clinical detection thresholds (ΔE≥2,7) ^19^.

## Material and methods

**Figure 1:**
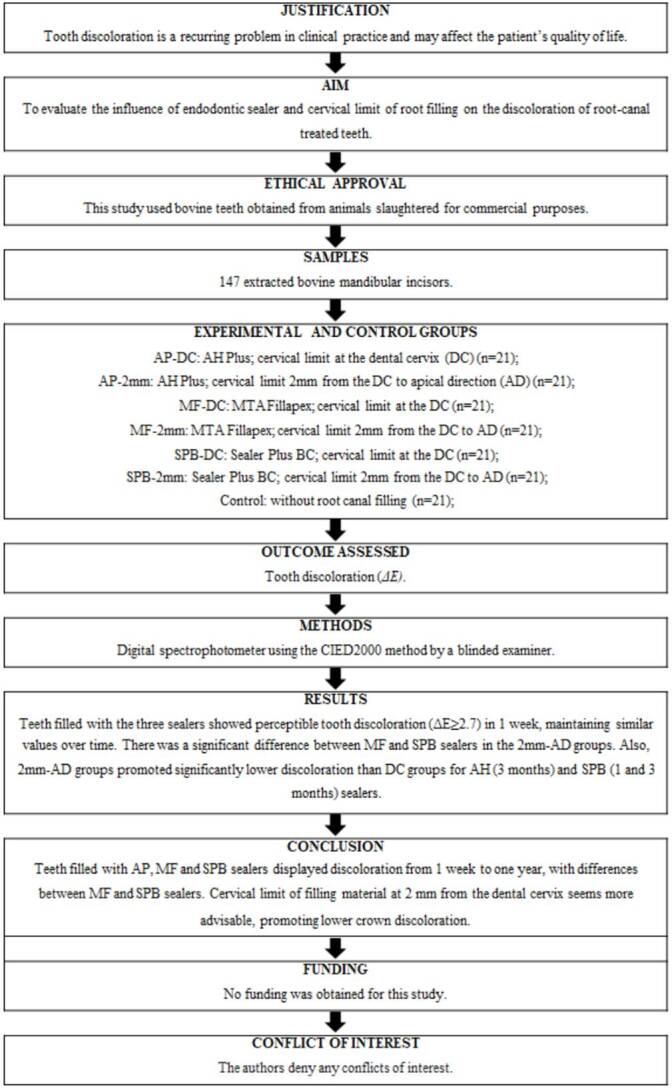
PPRILE 2021 Flowchart.

The manuscript was written based on the 'Preferred Reporting Items for Laboratory Studies in Endodontology (PRILE) 2021' guideline ^20^. The PRILE 2021 Flowchart is provided in Figure 1.

### Sample selection and preparation

The sample of this study was composed of bovine incisors from animals slaughtered for commercial purposes. The sample size calculation was performed based on the parameters described by Meincke et al.^21^: minimal difference between means of treatments was 2.50 (ΔΕ); standard deviation 2.10; power of the study 80%; α 0.05; for seven groups (BioEstat Program 5.0; Fundação Mamirauá, Belém, Brazil). The estimated minimum sample size was found to be 21 teeth per group, totaling 147 teeth, including six experimental groups and one control group.

The external root surfaces of the specimens were observed under a digital stereomicroscope (Stereo Discovery V20; Zeiss, Oberkochen, Germany) at 8x magnification. Teeth with extensive coronal wear, cracks or fracture lines, immature apices, external resorption, apical opening larger than a size 70 K-file (Dentsply Maillefer, Ballaigues, Switzerland), or other structural anomalies were excluded from the sample.

The root surfaces were cleaned with periodontal curettes (Golgran, São Paulo, Brazil) and with T1-S and T2-S inserts attached to a dental ultrasonic device (Sonic Laxis BP LED; Schuster, Santa Maria, RS, Brazil). After that, the bovine incisors were stored in distilled water, at 37º C, until use.

### Root canal treatment

The access cavity of all teeth was performed with spherical diamond bur 1016 (KG Sorensen, Cotia, SP, Brazil), followed by non-cutting tip bur 3082 (KG Sorensen, Cotia, SP, Brazil), booth mounted on a high-speed handpiece with water-cooling. Root canals were first irrigated with 5ml of sodium hypochlorite (NaOCl) 2,5% (Biodinâmica Química e Farmacêutica LTDA, Ibiporã, PR, Brazil). Next, a size #15 K-type file was inserted into the root canal until the tip could be seen through the apical foramen, and the working length was established by subtracting 1mm from this length.

During instrumentation and root canal obturation procedures, a physical barrier of utility wax (Clássico, Campo Lindo Paulista, SP, Brazil) was placed over the periapical apex of each tooth. Root canal preparation was performed with hand stainless steel files and Largo burs #4 and #3 (Dentsply Maillefer, Petrópolis, RJ, Brazil) were used for coronal pre-enlargement. The apical third was enlarged to a size #80 K-file (Dentsply Maillefer, Ballaigues, Switzerland). Intracanal irrigation was performed with 2 mL 2,5% NaOCl between each file. Then, the final rinse was performed with 2mL 17% EDTA (Asfer, São Caetano do Sul, SP, Brazil), and the apical file was used to agitate the solution. After 5 minutes, the residual effects of EDTA were removed by irrigating the canal with 2mL NaOCl, followed by the use of absorbent paper points (Dentsply Maillefer, Petrópolis, RJ, Brazil).

The specimens were randomized using the electronic tool www.randomization.com, and the teeth were assigned into six experimental groups and one control group (n=21), according to the endodontic sealers used and the cut-off level of the root canal obturation in the coronal area.

- AP-DC: AH Plus; cut at the dental cervix (DC);
- AP-2mm: AH Plus; cut 2mm from the DC to the apical direction (AD);
- MF-DC: MTA Fillapex; cut at the DC;
- MF-2mm: MTA Fillapex; cut 2mm from the DC to AD;
- SPB-DC: Sealer Plus BC; cut at the DC;
- SPB-2mm: Sealer Plus BC cut 2mm from the DC to AD;
- Control: This group received root canal instrumentation in the same way as the other groups, but it was maintained without root canal filling.

The main components of the tested materials are listed in Box 1. In all experimental groups, the root canal filling was performed by the lateral condensation technique. A size #80 gutta-percha master cone and accessory cones were used, allowing the endodontic sealer to come into contact with all root canal walls evenly until the leakage was visible through the pulp chamber. After radiographic confirmation of the root filling quality, the excess of material was removed with heated a nº1 Paiva condenser (Golgran, São Paulo, SP, Brazil), and final vertical compaction was completed with a nº2 Paiva condenser.

The cervical limit of the root filling was determined with the aid of a millimeter periodontal probing (S. S. White Duflex, Rio de Janeiro, RJ, Brazil), which was first inserted internally and then externally at the labial aspect of the tooth, to define the position of the cut at the DC, ie, at the CEJ (AP-C, MF-C, and SPB-C groups) or 2mm from the CEJ (AP-2mm, MF-2mm and SPB-2mm groups). Finally, the access cavities were cleaned using cotton pellets saturated with 95% ethanol (Ciclo Farma, Serrana, SP, Brazil), followed by the dental adhesive procedure.

All groups were sealed with composite resin. For that, the endodontic access was etched with 37% phosphoric acid (Biodinâmica, Ibiporã, PR, Brazil) for 15 seconds, rinsed with air-water spray for 30 seconds, and the excess moisture was removed with cotton pellets. Then, an adhesive system was applied and light-cured (Single Bond; 3M, Sumaré, SP, Brazil), followed by a composite resin restoration. The color of the material was A2 (Charisma; Kulzer, São Paulo, SP, Brazil) for all specimens, applied in small increments. A careful light-curing process was performed, with a calibrated light cure device and in different spots, ensuring an appropriate degree of conversion. The teeth were stored at 37º C in individual coded bottles and immersed in distilled water.

### Color change assessment (ΔE)

Color measurements were performed with a digital spectrophotometer (Easyshade Compact; VITA Zahnfabrik, Bad Saeckingen, Germany), following the guidelines from the International Commission on Illumination. The same examiner performed all color measurements in a dark room. This examiner was blinded for the experimental groups since each tooth received a code number. To standardize the color measurement location, a matrix was fabricated with an acetate plate (Essence Dental, Araraquara, SP, Brasil) in a vacuum plasticizer. A hole was made in this matrix, at the point corresponding to the cervical third of the buccal aspect of the crown, with the aid of a 6-mm spherical tungsten drill (American Burrs, Palhoça, SC, Brazil) mounted on a handpiece. That way, it was possible to determine the exact place for the spectrophotometer tip fitting. Three measurements were performed for each tooth (triplicate), and the median was calculated.

Colour measurements were carried out at the baseline (T0), before any intervention. After root canal treatment, the teeth were stored for 1 week in distilled water at 37ºC, and a second color measurement was performed (T1). The following evaluations were made at one month (T2), three months (T3), six months (T4) and 12 months (T5).

Color changes for each time interval were calculated, taking the baseline color measurement (T0) as a comparison (ΔE1, ΔE2, ΔE3, ΔE4 e ΔE5), considering the three dimensions of the system CIELab, where L* values describe lightness coordinate, ranging from 0 (black) to 100 (white), a* and b* are considered chroma coordinates: a* for red (+) and green (-) and b* for yellow (+) and blue (-) ^11^. For the calculation, the formulas CIEDE 2000, were used:

$$∆E_{00}=\sqrt{\left(\frac{∆L´}{K_{L}S_{L}}\right)+}\left(\frac{∆{C´}_{ab}}{K_{c}S_{c}}\right)+\left(\frac{∆{H´}_{ab}}{K_{H}S_{H}}\right)+R_{T}\left(\frac{∆{C´}_{ab}}{K_{c}S_{c}}\right)\left(\frac{∆{H´}_{ab}}{K_{H}S_{H}}\right)$$

### Statistical analysis

The data were expressed in means and standard deviation. The Shapiro-Wilk test demonstrated non-normal distributions of the values. Thus, the data were submitted to a logarithmic transformation to allow the use of parametric tests. ANOVA and Tuckey post hoc tests were used to evaluate the differences between the sealers and the cervical limits of root filling. Additionally, to assess the differences in color changes over time in each experimental group, ANOVA with repeated measures and Tuckey post-hoc tests were performed. All analyses were performed using SPSS statistics v.20 (SPSS Inc., Chicago, IL, EUA), and a significance level of 5% was assumed.

## Results

Table 1 shows the mean values of ΔE for the tested sealers during the follow-up periods. At ∆E1 (one week), the three endodontic sealers induced a clinically perceptible color change (ΔE≥2,7) with no differences between them and compared to the control (P>0.05). Regarding the cervical limit of root filling, in each sealer, there was no significant difference (P>0.05)

After one month (∆E2), there was a significant difference between MF and SPB sealers in the group of cervical limit at 2mm to apical direction (P=0.047). Regarding the cervical limit, the 2mm-AP level induced fewer color changes in comparison to the DC level; this finding was statiscally significant for the SPB sealer (P=0.02).

After three months (∆E3), no significant difference was detected between the three sealers and the control group (P>0.05). The color change at 2mm-AD was smaller than at the DC level; this difference was statiscally significant for AP (P=0.04) and SPB (P=0.01) sealers.

After six months (∆E4), no statistical difference was detected between the three sealers and the control group (P>0.05). Regarding the cervical limit, in each sealer, no significant difference was detected (P>0.05).

After one year (∆E4), no statistical difference was verified between the three sealers and the control group (P>0.05), and also between the cervical limits of root filling.

**Figure 2:**
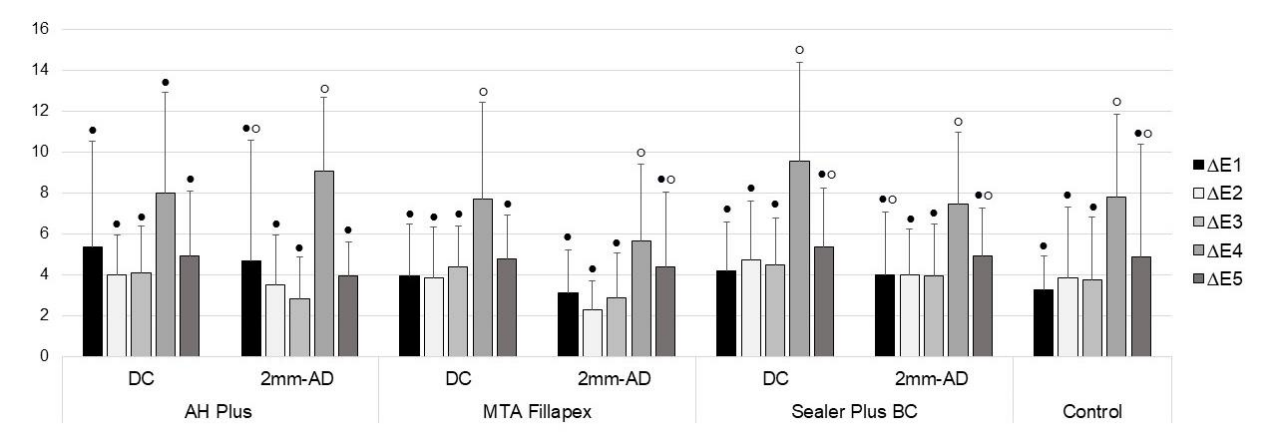
Graphic representation of color change for each experimental group over time. Different symbols (●/○) indicate statistical differences (α= 0.05), regarding the color changes over time, in each experimental group.

Figure 2 illustrates the colour changes over time, in each experimental group. The six-month assessment (∆E4) showed the highest averages of discoloration; this was statiscally significant for AP-2mm, MF-DC, MF-2mm, SPB-DC, SPB-2mm, and control groups.

**Table:** 1. Means and standard deviation (SD) values of ∆E for different experimental groups during the follow-up periods.

Sealer	Cervical limit	∆E1 Mean ± SD	∆E2 Mean ± SD	∆E3 Mean ± SD	∆E4 Mean ± SD	∆E5 Mean ± SD
AH Plus	Dental cervix	5.34 ± 5.21 ^A^	3.97 ± 1.96 ^A^	4.07 ± 2.31 ^A^	8.00 ± 4.90 ^A^	4.92 ± 3.18 ^A^
	2mm to apical direction	4.69 ± 5.89 ^a^	3.52 ± 2.40 ^ab^	2.80 ± 2.05 ^a^	9.06 ± 3.60 ^a^	3.95 ± 1.65 ^a^
	P	0.42	0.39	0.04*	0.88	0.62
MTA Fillapex	Dental cervix	3.93 ± 2.54 ^A^	3.84 ± 2.47 ^A^	4.37 ± 2.02 ^A^	7.68 ± 4.75 ^A^	4.76 ± 2.18 ^A^
	2mm to apical direction	3.12 ± 2.11 ^a^	2.29 ± 1.42 ^a^	2.89 ± 2.18 ^a^	5.67 ± 3.72 ^a^	4.37 ± 3.66 ^a^
	P	0.50	0.33	0.37	0.20	0.73
Sealer Plus BC	Dental cervix	4.18 ± 2.39 ^A^	4.73 ± 2.88 ^A^	4.50 ± 2.28 ^A^	9.57 ± 4.81 ^A^	5.38 ± 2.85 ^A^
	2mm to apical direction	3.99 ± 3.09 ^a^	3.99 ± 2.23 ^b^	3.92 ± 2.55 ^a^	7.48 ± 3.49 ^a^	4.99 ± 2.35 ^a^
	P	0.31	0.02*	0.01*	0.09	0.25
Control		3.28 ± 1.62 ^Aa^	3.87 ± 3.42 ^Aab^	3.74 ± 3.08 ^Aa^	7.80 ± 4.06 ^Aa^	4.86 ± 4.53 ^Aa^
SEALER		0.331 f=1.113	0.036 f=3.391	0.186 f=1.701	0.120 f=2.154	0.258 f=1.100
CUT-OFF LEVEL		0.154 f=2.050	0.020 f=5.510	0.002 f=9.889	0.076 f=3.200	0.347 f=1.290
CUT-OFF LEVEL*SEALER		0.970 f=0.030	0.561 f=0.580	0.488 f=0.721	0.538 f=0.622	0.838 f=0.177

Distinct uppercase letters represent significant differences between the sealers and control with the cervical limit at the dental cervix, within the same experimental period.

Distinct lowercase letters represent a significant difference between the sealers and control with the cervical limit at 2mm from the dental cervix, within the same experimental period.

*P value <0.05 represents a significant difference between the cut-off levels in each sealer.

## Discussion

The present study evaluated the influence of the endodontic sealer, including a new premixed calcium silicate-based material, and the cervical limit of root filling on the discoloration of root-canal-treated teeth. The null hypothesis was rejected since all tested sealers induced a clinically perceptible color change one week after obturation. To our knowledge, this is the first study to evaluate the tooth discoloration induced by the SPB sealer. However, previous investigations have already proved that other calcium silicate-based sealers induce relevant color changes in short periods ^12^. Regarding the potential to promote tooth discoloration of the gold standard endodontic sealer (AP), the findings are contradictory. Some authors report that AP did not promote clinically perceptible tooth discoloration in long periods ^14^ ^,^ ^15^, however, other studies demonstrated clinically perceptible tooth discoloration in ten days ^7^, two weeks ^22^, or one month ^12^.

There are also differences between the findings related to the MF sealer. The presence of MTA in its composition could be responsible for causing tooth discoloration ^2^. However, the amount of particles of MTA in MF seems minimal since the material presents other diverse resin components ^16^, which give it the right consistency to be used as an endodontic sealer. In addition, radiopacity is provided by bismuth oxide, which has been hypothesized to interact with the dentine collagen, resulting in a tooth with marked gray-colored alteration ^12^. Nonetheless, the radiopacifying agent was recently replaced by calcium tungstate (Box 1), a stable staining radiopacifier. According to Ioannidis et al.^11^, the MF sealer had minimal potential to promote a clinically perceptible tooth discoloration until three months after obturation, contrary to what happened in teeth obturated with a zinc oxide and eugenol-based sealer, which promotes severe and fast discoloration. On the other hand, other authors demonstrated that MF promotes ΔE values higher than the clinical detection threshold in only one month ^8^ ^,^ ^9^.

**Box 1:**
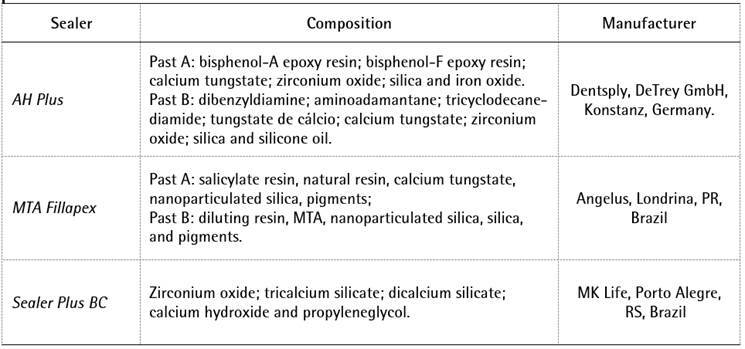
Description of the endodontic sealers used in the present study and their manufacturers.

The differences between the findings of tooth discoloration induced by endodontic sealers were probably related to the tremendous methodological difference in the studies. These differences could occur in terms of ^1^ Dentine substrate (human or bovine teeth); ^2^ Dental group (incisors, premolars, or molars); ^3^ Use of the crown or entire tooth; ^4^ Endodontic sealer associated or not to gutta-percha; ^5^ Pre-removal of *smear layer*. Regarding the presence or absence of gutta-percha, it is well known that it can lead to a ‘‘pink tone’’ in the crown ^6^, changing the coordinates a* (to red) and b* (to yellow) of the CIELab system ^23^. In addition, the presence of *a smear layer* drastically reduces the dentinal tubule penetration ^24^, which could influence the diffusion of pigments. This can be observed in studies where the root canal obturation is performed with no attempt to remove the *smear layer*. In these cases, the tooth discoloration seems less evident or takes more time to occur ^3^.

Another factor that could be responsible for the differences in the findings of tooth discoloration is the method used to evaluate the color change. The CIE (Commission International de l’Eclairage) recommends different color notation systems to assess color change ^25^. In the endodontic field, the majority of studies regarding tooth discoloration induced by endodontic sealers use the CIELAB coordinates to determine tooth discoloration ^5^ ^,^ ^9^ ^,^ ^11^ ^,^ ^23^. However, the CIELAB is not the best method to calculate color discrepancy ^25^. Previous studies ^26^ about the perceptibility and acceptability of color differences demonstrated discrepancies in the sensitivity of the coordinates (L*), (a*), and (b*). Also, CIELAB has a limited color space, making it difficult to assess small color changes ^27^. According to Pecho et al. (2016) ^25^, the CIEDE method should be recommended in studies about color differences. The CIEDE formula comes closer to human perceptibility and acceptability in assessing the difference between dental shades, and also it considers the interactions and differences in chroma and hue ^25^. Due to this methodological heterogeneity between the studies, our findings should be carefully compared with others; nonetheless, both formulas use the same parameters and are strongly correlated ^28^.

In the present study, the control group promoted color change similar to the groups of teeth obturated with gutta-percha and endodontic sealer, contrary to what was observed by other authors ^7^ ^,^ ^8^ ^,^ ^15^ ^,^ ^23^. It is important to point out that in these previous investigations, an empty pulp chamber was used as control, while in our study the control group was composed of teeth that received root canal instrumentation but were not filled. Also, the endodontic access was restored with composite resin, as previously described ^22^. The aim here was to isolate the influence of root canal obturation on tooth discoloration. The results of our study demonstrated that the presence of a restorative material, sealing the coronal cavity, already promotes color changes in the dental crown; these findings are in agreement with Elkhazin ^22^.

One may argue that the ∆E values decreased considerably from ∆E4 to ∆E5; however, this is not surprising once similar results had already been described ^29^ ^,^ ^30^. A possible explanation for this decline could be attributed to alterations in the tooth structure’s optical proprieties related to the endodontic sealer’s physical presence in contact with the dentine and the consequent interaction of the resinous matrix with the dentinal surface ^30^.

The evaluation of the cervical limit of root filling demonstrated that the 2mm-AD level promoted a minor color change in comparison to the DC level when different sealers and times of follow-up were assessed. This finding was already expected and confirms the need for a careful definition of the cervical limit to cut off the root-filling material. The dentine substrate is composed of dentinal tubules that are the track between the internal structures (pulp cavity) and the outer face of the tooth ^31^. Therefore, the cervical limit of root filling must maintain a safety margin to reduce the chances of tooth discoloration. Although the recommendation for the pulp chamber cleaning after root canal obturation is the use of 95% ethanol ^10^, it has already been demonstrated that alcohol and other different protocols are not efficient for removing remnants from endodontic sealers ^32^.

Within the limitations of this study, bovine teeth were used to assess the influence of endodontic sealers and the cervical limits on tooth discoloration. It is important to point out that bovine teeth have been extensively used to evaluate the potential of tooth discoloration caused by endodontic materials ^15^ ^,^ ^33^ ^,^ ^34^. The use of human teeth for *in vitro* studies is limited by ethical reasons and because it is getting harder and harder to get specimens caries-free or without restorations ^34^. Besides that, using bovine dentine samples brings some advantages related to standardization since it is possible to obtain several teeth from a few animals, which minimizes confounding factors like tooth age, occlusal condition, and diet ^35^. Although bovine dentine has a higher density of dentinal tubules, the coronal dentine layers did not differ significantly in terms of density and diameter of tubules (36), which suggests that the human crowns can be replaced by bovine crowns in laboratory studies ^15^, especially since human extracted teeth are increasingly difficult to obtain for laboratory research.

Tooth discoloration was assessed only in the cervical third because this is the region most affected by teeth discolored by endodontic materials ^5^ ^,^ ^6^. According to a previous investigation from Partovi et al. ^5^, when the color change was evaluated in premolars, the chromatic changes were higher at the cervical segment of the root and the cervical third of the crown, with minimal changes in the occlusal third. One possible explanation is that particles from the endodontic sealer are spread by the dentinal tubules in the direction of enamel, a colorless and translucent structure. Once in the cervical third, the enamel is thinner, and the discoloration becomes more evident.

Although carefully designed, this *in vitro* study is only an estimate of possible tooth discoloration promoted by resin and calcium silicate sealers. The findings must be confirmed by long-term prospective clinical trials since different mechanisms of darkening can occur in the *in vivo* scenario, such as the interaction of endodontic materials with salivary and bacterial components, in cases of infiltration at the margins of restorations ^15^. Nonetheless, the clinical significance of the present study lies in the fact that the chromogenic potential of the endodontic sealer may play a crucial role in selecting the appropriate endodontic material during the filling procedures. Furthermore, our findings reinforce the influence of different root canal sealers on tooth discoloration, especially given that one of these sealers had not been previously tested, and highlight the importance of placing the cervical limit of root filling material 2mm away from the dental cervix.

In conclusion, teeth filled with AP, MF, and SPB sealers displayed discoloration from 1 week to 12 months, and there were statistical differences between MF and SPB sealers at the one-month assessment. The cervical limit of root filling at 2 mm in the apical direction seems more advisable, promoting lower crown discoloration. Besides that, the period of six months demonstrated higher color change.

## Data Availability

The data that support the findings of this study are available from the corresponding author upon reasonable request.
